# Supplementary material for: Identification of photoperiod-induced specific miRNAs in the adrenal glands of Sunite sheep (Ovis aries)
Source: Front Vet Sci. 2022 Jul 22;9:888207. doi: 10.3389/fvets.2022.888207 (PMC9354845; doi:10.3389/fvets.2022.888207)
Supplement: Supplementary file 1 [file Data_Sheet_1.ZIP › Supplementary file/miRDeep2 file.zip!/miRDeep2 file/Novel_Structure/SPLP42A3/Novel_218.pdf]

Provisional ID	:	NC_019478.2_33536
Score total	:	1.9
Score for star read(s)	:	-1.3
Score for read counts	:	0
Score for mfe	:	1.6
Score for randfold	:	1.6
Score for cons. seed	:	
Total read count	:	8521
Mature read count	:	8521
Loop read count	:	0
Star read count	:	0

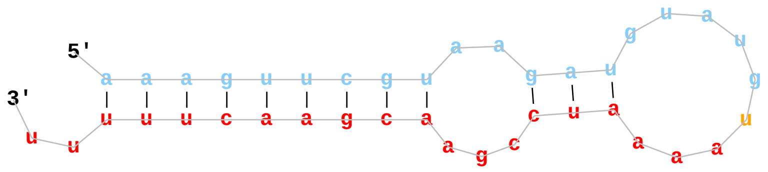

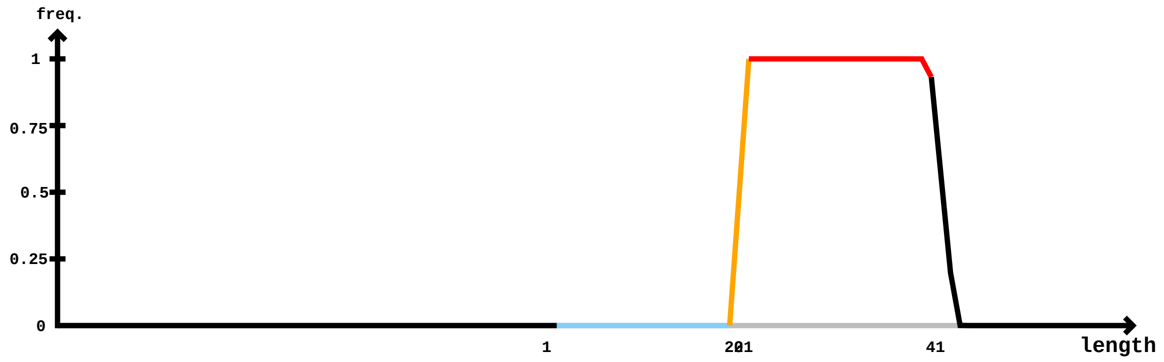

Star **Mature**

```
5'- gcagaggaaggaaggagggggugucugagaggguauugggcuugggccagaaaguucguaagauguauguaaaauccgaacgaacuuuuugggcccacccaguauuucaa   -3'   exp
    .((((.(..............).))))...((((((((((..(((((((((((((((((..(((.........)))...))))))))))).))))))))))))))))...   reads   mm   sample
    .....................................................................uaaaauccgaacgaacuuuuu....................   2       0    seq
    ......................................................................aaaauccgaacgaacuu.......................   1       0    seq
    ......................................................................aaaauccgaacgaacuuuu.....................   579     0    seq
    ......................................................................aaaauccgaacAaacuuuuu....................   6       1    seq
    ......................................................................aaaauccgaacgaacuuuuu....................   6243    0    seq
    ......................................................................aaaauccgaacAaacuuuuug...................   1       1    seq
    ......................................................................aaaauccAaacgaacuuuuugg..................   2       1    seq
    ......................................................................aaaauccgaacgaacuuuuugg..................   1683    0    seq
    ......................................................................aaaauccgaacgaacuuuuuggg.................   4       0    seq
```
